# Prevalence of psychological distress: a scientific approach towards the mental health and wellbeing of population during the SARS-COV-2 outbreak

**DOI:** 10.12688/f1000research.145483.2

**Published:** 2024-10-21

**Authors:** Saman Tauqir, Inayat Shah, Ahmed Alsubaie, Sara Noreen, Shazia Sadaf, Saqib Ali

**Affiliations:** 1 Department of Physiology, Institute of Basic Medical Sciences, Khyber Medical University, Peshawar, Pakistan; 2 Department of Biomedical Dental Sciences, College of Dentistry, Imam Abdulrahman Bin Faisal University, P.O. Box 1982, Dammam 31441, Saudi Arabia; 3 Department of Medicine, Khyber Teaching Hospital, Peshawar, Pakistan; 4 Department of Dental Education, College of Dentistry, Imam Abdulrahman Bin Faisal University, P.O. Box 1982, Dammam 31441, Saudi Arabia

**Keywords:** COVID-19, Kesseler scale, SARS-CoV-2, Mental health, Pandemic, Pakistan

## Abstract

**Background:**

The global challenge of the novel coronavirus has led to an unprecedented downturn, adversely affecting the health and mental wellbeing of communities worldwide. The objective of this study is to assess mental health and psychological distress levels within the general population of Peshawar, Pakistan amidst the ongoing COVID-19 pandemic.

**Method:**

A cross-sectional online survey was conducted among 715 residents of Peshawar, Pakistan. The questionnaire collected data on demographics, socioeconomic status, and residential area. The Modified K10 Kessler Psychological Distress Scale was used to measure mental well-being. Descriptive statistics, including means and standard deviations, were calculated, and statistical analyses were performed using SPSS (IBM, USA, version 22).

**Results:**

A total of 715 responses were collected. The comprehensive psychological distress score was identified as 25.55, signifying moderate stress levels. Among the respondents, 53.3% were females, 46.7% had completed a bachelor’s degree, 41% were employed, 78.7% were single, 93.1% were non-smokers, and 69.4% resided in urban areas. The study revealed that both gender (p=0.001) employment status (p=0.018) were linked to a more pronounced psychological impact of the outbreak.

**Conclusions:**

This study highlights age, gender, employment status, and urbanization as influential factors contributing to psychological distress during the COVID-19 outbreak. As we face the challenges of the new normal, it is essential for policymakers to address these mental health concerns through targeted support and interventions, ensuring that mental health resources are accessible and responsive to the needs of affected populations.

## Inroduction

The emergence of Coronavirus Disease 2019 (COVID-19) in Wuhan, China, in December 2019 swiftly escalated into a global pandemic within months. ^1^ The consequences of this public health crisis have significantly impacted the mental well-being of the population, giving rise to a surge in psychological crises. ^2^^,^^3^ Identifying populations experiencing early-stage psychological crises is crucial for the effective implementation of intervention strategies. Despite a widespread increase in mental distress reported among both the general public and frontline medical personnel, the specific determinants of psychological distress remain unidentified across the diverse populations affected by the COVID-19 pandemic. ^2^^–^^4^

Historically, pandemics have had profound impacts on mental health. For example, the Spanish Flu of 1918, the SARS outbreak in 2003, and the Ebola epidemic in 2014 led to significant psychological challenges, including increased anxiety, stress, and trauma due to fear of infection, high mortality rates, and societal disruption. Traumatic public health emergencies can induce feelings of insecurity, driven by the fear of infection and mortality. Symptoms such as anxiety, stress, insomnia, and fear are frequently observed during pandemics. ^5^ Various studies conducted during the pandemic have reported increased psychological distress in the general population and a significant proportion of healthcare professionals. ^6^^,^^7^ The fear of infection, economic downturn, job losses, vaccination and reduced income due to prolonged lockdowns are collectively contributing to mental health disorders in society, ultimately leading to depression and suicidal thoughts. ^3^^,^^8^^,^^9^

The mental health implications of the COVID-19 pandemic are of paramount importance, affecting the general population on multiple fronts. ^10^^,^^11^ Our frontline heroes, including all medical professionals directly engaged with COVID-19-infected and quarantined patients, endure constant and substantial psychological trauma. ^10^ Stress predictors for these individuals include persistent fears of contracting the virus, overwhelming workloads, and the emotional toll of witnessing COVID-19 patients passing away in isolation. ^11^

It is crucial to acknowledge and address the psychological traumas and mental health challenges faced by individuals in the wake of the pandemic. Implementing measures like lockdowns, while necessary for control, can induce anxiety responses and contribute to increased fear and prejudice against those infected or affected. ^12^ Studies examining the impact of COVID-19 on mental well-being not only shed light on critical areas of concern but also offer insights into how healthcare services can be equipped with essential information and support to provide mental health treatment to those in need. ^13^ Consequently, prioritizing public mental health is paramount, necessitating the adaptation of policies to support individuals in navigating the challenges posed by the “new normal.” The primary objective of this study is to assess mental health and psychological distress levels within the general population of Peshawar, Pakistan amidst the ongoing COVID-19 pandemic.

## Methods

### Study design

The research design employed for this study was cross-sectional, conducted among the population of Peshawar city Pakistan using an online survey. The survey was distributed to the public through diverse social platforms, including WhatsApp, Twitter, emails, and Facebook messengers.

### Ethics

Approval for ethical considerations was granted by the Ethics Review Committee of the Khyber Medical University (approval number: DIR/KMU-EB/PS/000109, granted on 02-02-2022). This study was conducted conferring to declaration of Helsinki. Written informed consent was obtained from participants via a consent statement for participation incorporated before the survey, stating, “Your involvement in this study is entirely voluntary. There are no anticipated risks associated with this project. However, if you find any questions uncomfortable, you have the option to withdraw from the survey at any point.” Thus, participation in the survey was considered as implicit consent to participate.

### Participants

Survey participants encompassed individuals of both genders, aged between 15 and 60, and representing various educational backgrounds. The study targeted individuals with internet access, encompassing a diverse group, including undergraduate students, engineers, medical professionals, government employees, retirees, lawyers, business professionals, and individuals from various other professions. Prior to survey completion, informed consent was obtained from all participants, and their responses were kept confidential and anonymous. The survey took approximately five to seven minutes to finish.

### Data collection

Data collection for this study utilized an online survey conducted through Google Forms. The questionnaire for this study was structured into sections, covering demographic characteristics (gender, education level, marital status, job status and area of residing whether urban or rural).

For assessing distress, K10 was used which serves as a concise screening method to assess various levels of distress. The K10 scale was selected due to its strong validity and reliability in measuring psychological distress across diverse populations and its sensitivity to detecting symptoms of anxiety and depression. The Kessler Psychological Distress Scale (K10) is a straightforward tool for gauging psychological distress. ^14^ Comprising 10 questions regarding emotional states, each with a five-level response scale.

### Scoring criteria

Scoring for each item ranges from one, indicating ‘none of the time,’ to five, indicating ‘all of the time.’ The scores for the 10 items are then totaled, resulting in a minimum score of 10 and a maximum score of 50. Lower scores suggest minimal psychological distress, while higher scores indicate elevated levels of psychological distress.

In terms of score interpretation, cut-off scores as a reference for screening psychological distress are as follows: ^15^

K10 Score: Likelihood of having a mental disorder (psychological distress)

10-19: Likely to be well

20-24: Likely to have a mild disorder

25-29: Likely to have a moderate disorder

30-50: Likely to have a severe disorder.

### Statistical analysis

Descriptive statistics, including mean and standard deviations, were utilized for data analysis. Statistical analyses were performed using Statistical Package for Social Science (SPSS, IBM USA,version 22), applying one-way ANOVA based on the number of groups and items to be compared. Results were presented as means and ± Standard Deviation SD, and differences were deemed statistically significant if p≤0.05. This comprehensive methodology ensured a rigorous and systematic approach to understanding the mental health dynamics in the specified population during the challenging times of the COVID-19 pandemic.

## Results

### Participant sociodemographic characteristics

The study included 715 respondents, reflecting a diverse participant profile ( Table 1). The overall psychological distress score averaged 25.55, indicating a moderate level of stress. Key sociodemographic characteristics revealed that the majority of participants were female (53.3%), held a bachelor's degree (46.7%), were employed (41%), single (78.7%), non-smokers (93.1%), and resided in urban areas (69.4%).

**Table 1.** Showing the demographics with percentages and frequencies.

Demographics		Percentage %	Number of respondents n
**Age**	15-20	32%	229
	21-30	50.6%	336
	31-40	12%	86
	41-50	3.6%	26
	51-60	1.3%	9
	60 and above	0.4%	3
**Gender**	Male	46.7%	334
	Female	53.3%	381
**Location**	Urban	30.6%	218
	Rural	69.4%	495
**Smoking**	Smoker	6.9%	49
	Non-smoker	93.1%	666
**Marital status**	Single	78.7%	563
	Married	20.1%	144
	Widowed	0.7%	5
	Divorced	0.4%	3
**Profession**	Doctor	21.7%	155
	Engineer	1.8%	13
	Lawyers	2.8%	20
	Business person	3.1%	22
	Unemployed	5.3%	38
	Retired	1%	7
	Government service	1.3%	9
	Private job	4.2%	30
	Student	58%	415
	Unemployed due to COVID-19	0.8%	6

### Psychological responses during the lockdown

Table 2 presents the psychological responses of the respondents to the Kessler scale during the lockdown in Peshawar, Pakistan. Analyzing the data from Table 2, it is observed that 22.7% (162 respondents) reported feeling tired most of the time in the last month, with 7% (50 respondents) expressing constant fatigue over the 30-day period. Approximately 15.9% (114 respondents) noted feeling nervous most of the time during the pandemic, while 30.3% (217 respondents) experienced occasional nervousness. Conversely, 2.7% (19 respondents) reported constant nervousness throughout the pandemic. Furthermore, 10.1% (72 respondents) felt uncalmable during the past month, whereas 41.4% (296 respondents) indicated a sense of calmness, suggesting adaptation to the new normal. Unfortunately, 6.6% (47 respondents) felt hopeless about the ongoing situation, while 35.5% (254 respondents) remained hopeful for improvement. Additionally, 31% (222 respondents) experienced restlessness at times, and 25.9% (185 respondents) felt so restless that they could not stand still. Finally, 19.6% (140 respondents) reported feeling depressed most of the time, and 7.7% (55 respondents) expressed constant depression due to the outbreak of the viral disease. These findings provide a comprehensive understanding of the psychological responses of the participants during the specified period, shedding light on the multifaceted impact of the COVID-19 pandemic on mental well-being.

**Table 2.** Participants' Responses to Kessler’s Questions on a 5-Point Likert Scale (1 none of the time) to (5 all of the time).

SNO.	Parameters tested	None of the time (n)%	A little of the time (n)%	Some of the time (n)%	Most of the time (n)%	All of the time (n)%
1	How often did you feel tired out for no good reason?	(131) 18.3%	(162) 22.7%	(210) 29.4%	(162) 22.7%	(50) 7%
2	How often did you feel nervous?	(157) 22%	(208) 29.1%	(217) 30.3%	(114) 15.9%	(19) 2.7%
3	How often did you feel so nervous that nothing could calm you down?	(296) 41.4%	(193) 27%	(139) 19.4%	(72) 10.1%	(15) 2.1%
4	How often did you feel hopeless?	(254) 35.5%	(161) 22.5%	(146) 20.4%	(107) 15%	(47) 6.6%
5	How often did you feel restless or fidgety?	(139) 19.4%	(222) 31%	(195) 27.3%	(128) 17.9%	(31) 4.3%
6	How often did you feel so restless you could not sit still?	(303) 42.2%	(185) 25.9%	(137) 19.2%	(70) 9.8%	(20) 2.8%
7	How often did you feel depressed?	(149) 20.8%	(198) 27.7%	(173) 24.2%	(140) 19.6%	(55) 7.7%
8	How often did you feel that everything was an effort?	(138) 19.4%	(165) 23.1%	(182) 25.5%	(157) 22%	(72) 10.1%
9	How often did you feel so sad that nothing could cheer you up?	(194) 27.1%	(20.8) 29.1%	(173) 24.2%	(103) 14.4%	(37) 5.2%
10	How often did you feel worthless?	(238) 33.3%	(186) 26%	(152) 21.3%	(90) 12.6%	(49) 6.9%

### Psychological distress measures

Table 3 presents the mean values and corresponding standard deviations of each question on the Kessler Scale 10, which measures psychological distress. The data provides insights into the frequency and intensity of various stress-related parameters among the surveyed individuals in Peshawar, Pakistan during the COVID-19 pandemic. Additionally, the table includes the 95% confidence intervals of the mean differences, offering a statistical perspective on the reliability of the reported values.

**Table 3.** Mean Values of each question of Kessler Scale 10.

SNO.	Parameters tested	Mean	± SD	95% CI of diff
1	How often did you feel tired out for no good reason?	2.773	1.190	-1.935 to -1.612
2	How often did you feel nervous?	2.483	1.081	-1.644 to -1.321
3	How often did you feel so nervous that nothing could calm you down?	2.045	1.095	-1.207 to -0.8829
4	How often did you feel hopeless?	2.345	1.278	-1.507 to -1.184
5	How often did you feel restless or fidgety?	2.566	1.120	-1.728 to -1.405
6	How often did you feel so restless you could not sit still?	2.566	1.120	-1.728 to -1.405
7	How often did you feel depressed?	2.656	1.224	-1.818 to -1.494
8	How often did you feel that everything was an effort?	2.801	1.262	-1.963 to -1.640
9	How often did you feel so sad that nothing could cheer you up?	2.414	1.177	-1.576 to -1.252
10	How often did you feel worthless?	2.337	1.247	-1.499 to -1.175

CI=Confidence Interval, SD= Standard Deviation.

### Demographic determinants of stress

A comparison of Kessler Scores across different demographic factors, exploring the determinants of stress among the surveyed individuals is presented in Table 4. It shows the mean values, standard deviations, and p-values for various demographic categories. The Kessler scores of females were found to be significantly higher than those of male participants (27.36 vs. 23.48, p = 0.001). Similarly, employed participants exhibited higher scores compared to their non-employed counterparts (25.72 vs. 22.47, p = 0.018), suggesting potential variations in stress levels based on gender and employment status Table 4.

**Table 4.** Comparison of Kessler Scores Across Demographic Factors.

Determents of stress		Mean	SD	P-value
Gender	Male	23.4880	7.90663	0.001
	Female	27.3622	8.19853	
Living	Urban	25.8444	8.29114	0.127
	Rural	24.8165	8.23745	
Employment	Employed	25.7253	8.34065	0.018
	Unemployed	22.4737	6.62402	

SD= Standard Deviation.

This table provides an overview of the demographic distribution of survey participants, including percentages and frequencies. The categories encompass age groups, gender, location, smoking habits, marital status, and profession. The legend offers a comprehensive understanding of the respondents' diverse characteristics and backgrounds.

This table presents the detailed responses of participants to Kessler’s questions, each assessed on a 5-point Likert scale ranging from “None of the time” to “All of the time.” The parameters tested include feelings of tiredness, nervousness, hopelessness, restlessness, depression, and worthlessness. The legend provides a breakdown of participant responses across various levels of frequency for each parameter, offering insights into the mental well-being of the surveyed individuals.

This table displays the mean values and standard deviations of each question in the K10 assessment. The parameters tested include feelings of tiredness, nervousness, hopelessness, restlessness, depression, and worthlessness. Additionally, the 95% confidence intervals of the differences are provided, offering insights into the variability of responses and the overall mental health assessment based on the participants' mean scores for each parameter.

Table 4 reveals that females Kessler score was significantly higher than male participants (27.36 vs 23.48, p – 0.001). Similarly, the score was higher in employed participants (25.72 vs 22.47, p – 0.018). However, living in Urban and Rural site (25.84 vs 24.81, p – 0.127).

## Discussion

In our cross-sectional study, we sought to dissect the effects of the COVID-19 pandemic on the mental health of the population in Peshawar Pakistan. The study's results reveal a significant impact of the COVID-19 pandemic on the mental health of individuals in Peshawar, Pakistan. The detailed psychological responses during the lockdown underscore the multifaceted nature of the pandemic's impact, encompassing fatigue, nervousness, feelings of hopelessness, and varied experiences of restlessness and depression.

The study reports an overall psychological distress score of 25.55 suggesting moderate level of stress among the diverse group of 715 respondents. Notable gender disparities were observed, with females exhibiting significantly higher distress scores than males (27.36 vs 23.48, p=0.001) ( Table 4). Employed individuals demonstrated higher distress scores (p =0.018) ( Table 4). In line with our results a studies in Nepalian populations and population of the United Kingdom reports high stress during the pandemic suggesting that there is a crucial need to carry out psychological interventional programs to cope with the situation effectively. ^16^^,^^17^

Our study reports the stress score of 27.36 in females indicating moderate stress levels. These findings are in line with the findings of a study conducted by Farooq et al. ^18^, revealing that females exhibited 2.5 times higher levels of stress compared to males (39.4% vs. 23.3%, respectively) ( Table 4). Another investigation found that the prevalence of depressive symptoms were 66% among women, contrasting 33% among men. ^19^ Globally conducted studies, including those in China, ^1^ India, ^20^ and Spain, ^21^ support these findings by reporting elevated stress symptoms among females. Recently Qiu et al., reported from China that the female gender and younger population were at a higher risk for different mental health outcomes and suffered from anxiety and stress. ^22^^,^^23^ These findings are also in line with another cross-sectional study carried out in Turkey which also reported that the group most affected psychologically by the pandemic were females, and the urban population. Priority might therefore be attached to these in future psychiatric planning. ^5^ Similarly, an Iranian study also reported the high prevalence of stress and depression among the females (95%) of Iranian during the pandemic. ^24^ Plausible explanations for the heightened prevalence of stress in women include biological factors, socioeconomic disadvantages, a loss of social status, maladapted coping strategies, and a lack of support systems in the country. ^25^ Additionally, socio-cultural norms in Pakistani households often lead women to juggle both household and professional responsibilities, while men are typically less involved in domestic activities. The ‘stay home, stay safe’ policy during the pandemic has intensified the workload for women as men spend more time at home.

These findings could be of great use in proposing new policies for global mental health related problems during the new normal.

Employment status also played a role, as employed individuals demonstrated higher distress scores compared to their unemployed counterparts (25.72 vs 22.47, p =0.018) ( Table 4). Anticipated salary cuts and a decline in job opportunities, coupled with heightened uncertainty and potential fear, may contribute to an increase in stress. ^12^ Furthermore, the closure of offices due to travel restrictions has resulted in a shift to remote work for most employees. The lack of in-person interaction with colleagues could impact workers' motivation, job satisfaction, and overall productivity. ^12^^,^^26^ The inability to meet deadlines and achieve targets, compounded by the added responsibilities of managing household affairs, may elevate anxiety levels among employees. ^12^

The SARSCoV-2 2019 is a highly contagious virus spreading via droplets or direct contact. Such viruses are transmitted easily in urban areas compared to rural areas with thick populations. ^27^ Therefore, a higher prevalence of mental distress is evident in both urban and semi-urban settings. This may be attributed to the increased incidence of COVID-19 in urban areas, where the lockdown has significantly impacted densely populated cities throughout Pakistan, effectively putting the lives of their residents at risk. ^28^^,^^29^ Our study reports a greater psychological impact of the virus on the individuals living in urban areas 25.8 ( Table 4) having access to communication and latest chaos caused by pandemic reporting. The results are corroborated by a Chinese investigation that demonstrated a higher occurrence of psychological health issues among individuals residing in urban areas. This trend is attributed to the substantial number of COVID-19 cases concentrated in cities, with urban areas serving as epicenters of the disease. ^30^

Developing countries face different challenges providing e-mental health care services due to lack of useful online interventions. ^31^ Responsible organizations of the country should design and enforce a gender-sensitive psychosocial protocol in form of videos or phone calls to ensure mental wellbeing in the nation. NGOs are efficient and effective mode to be used in this regard because of their close relation with communities, therefore they should be brought to proper use in this area.

## Conclusion

In conclusion, our cross-sectional study in Peshawar, Pakistan, reports a moderate overall psychological distress score of 25.55 indicates a noteworthy level of stress among the diverse group of 715 respondents. Notable gender disparities highlight that females exhibit significantly higher distress scores than males. Employment status also plays a role, with employed individuals demonstrating higher distress scores. Urban areas, acting as epicenters of the disease, experience a higher prevalence of mental distress, emphasizing the urban-rural divide in the impact of the virus. Our study provides a platform for the policy health care policy makers of our country to prioritize mental health issues in the time of new normal.

### Limitations

While our study provides valuable insights into the psychological state during the “new normal,” it is essential to acknowledge its limitations. The survey's cross-sectional nature limits our ability to make conclusions regarding the long-term effects of the pandemic, representing a notable study limitation. Additionally, potential selection bias exists as the survey relied on an online questionnaire, excluding individuals without Internet access or those unable or unwilling to use smartphones and emails. To improve generalizability in future studies, we recommend complementing online surveys with alternative distribution methods, such as paper surveys or in-person interviews, especially in settings where internet access is limited. By acknowledging these limitations, we aim to provide a balanced interpretation of our findings and offer suggestions for future research to mitigate these biases.

### Recommendations

Future research should focus on healthcare workers and their families to further explore the profound mental health impacts within this high-risk group. To support these individuals, it is essential to implement comprehensive psychological support measures. We recommend developing a range of psychological intervention programs accessible through various mediums, such as web videos, e-messages, advertisements, and dedicated mental health helpline services. These resources can promote resilience and provide timely mental health support throughout the COVID-19 pandemic.

The government should act swiftly to introduce e-mental health services to reduce mental stress. This can include telehealth counseling, online stress management resources, and interactive mental wellness platforms. Additionally, resilience-building programs, such as resilience training and stress management workshops, can be implemented to equip healthcare workers and the general public with coping strategies for navigating challenging times.

In the workplace, policies that encourage reasonable work hours, regular breaks, and adequate leave days are essential for reducing stress and supporting overall well-being among healthcare staff. Healthcare institutions and policymakers should prioritize these changes to address and prevent psychological distress effectively, promoting a healthier, more resilient workforce equipped to face future challenges.

By implementing these targeted mental health resources and policy measures, we can foster a supportive environment that enhances both individual and community resilience during and beyond the pandemic.

## Data Availability

Figshare: Prevalence of psychological distress: A Scientific approach towards the mental health and wellbeing of population during the SARS-COV-2 outbreak. https://doi.org/10.6084/m9.figshare.24638121.v1. ^32^ This project contains the following underlying data:
• data (2).xlsx Figshare: Prevalence of psychological distress: A Scientific approach towards the mental health and wellbeing of population during the SARS-COV-2 outbreak. https://doi.org/10.6084/m9.figshare.24638121.v1. ^32^ This project contains the following extended data:
• kessler.pdf (copy of questionnaire) Data are available under the terms of the Creative Commons Attribution 4.0 International license (CC-BY 4.0).
