# Supplementary material for: Prevalence of psychological distress: a scientific approach towards the mental health and wellbeing of population during the SARS-COV-2 outbreak
Source: F1000Res. 2024 Oct 21;13:339. Originally published 2024 Apr 23. [Version 2] doi: 10.12688/f1000research.145483.2 (PMC11809635; doi:10.12688/f1000research.145483.2)
Supplement: Peer review history

# Reviewer report: Reviewer response for version 2

**DOI:** 10.5256/f1000research.173331.r355153

**Published:** 2025-01-07

**Authors:** Gordana Stankovska

**Affiliations:** 1 University of Tetovo, Tetovo, North Macedonia

This study is very interesting, challenging and topical problem.The authors on a very simple, consistent  and clear way explain the relationship between psychological distress and well-being among respondents of Pakistan during the  SARS-COV-2 outbreak. Also they recommended that the support and psychological intervention programs can promote resilience and good well-being of person in every type of crises.

Is the work clearly and accurately presented and does it cite the current literature?

Yes

If applicable, is the statistical analysis and its interpretation appropriate?

Yes

Are all the source data underlying the results available to ensure full reproducibility?

Yes

Is the study design appropriate and is the work technically sound?

Yes

Are the conclusions drawn adequately supported by the results?

Yes

Are sufficient details of methods and analysis provided to allow replication by others?

Yes

Reviewer Expertise:

mental disorder, mental health, psychological counseling

I confirm that I have read this submission and believe that I have an appropriate level of expertise to confirm that it is of an acceptable scientific standard.

# Reviewer report: Reviewer response for version 1

**DOI:** 10.5256/f1000research.159438.r272711

**Published:** 2024-06-11

**Authors:** Aziza Alam

**Affiliations:** 1 Department of Community Medicine, Khyber Medical College, Peshawar, Pakistan

The presented study briefly encapsulates the essence of your study on the assessment of mental health and psychological distress within the general population of Peshawar, Pakistan, during the COVID-19 pandemic. Here are some suggestions and comments for potential refinement and further consideration:

 1.    Introduction: It's concise and effectively sets the stage for the study's relevance. You might want to add a brief note on why Peshawar specifically was chosen, if there's a unique context that makes this study particularly important for the region.

 2.    Expand Background Context: Provide more background on the mental health impacts of pandemics historically or from recent events prior to COVID-19. This could help frame the significance of your study within a broader context.

 3.    Method: This section is clear about the study's design and tools used for data collection. It could be enhanced by briefly mentioning the rationale behind using the Kessler Psychological Distress Scale specifically, which could help non-specialist readers understand its relevance and validity in this context.

 4.    The conclusion rightly emphasizes the implications for policy. It could be strengthened by explicitly recommending specific policy measures or interventions that could be implemented based on the study findings.

 5.    Address Bias and Generalizability in your limitations. More thoroughly discuss how the method of survey distribution (online) might limit the generalizability of the findings. For instance, does this method potentially exclude older populations or those without internet access?

Is the work clearly and accurately presented and does it cite the current literature?

Yes

If applicable, is the statistical analysis and its interpretation appropriate?

Yes

Are all the source data underlying the results available to ensure full reproducibility?

Yes

Is the study design appropriate and is the work technically sound?

Yes

Are the conclusions drawn adequately supported by the results?

Yes

Are sufficient details of methods and analysis provided to allow replication by others?

Yes

Reviewer Expertise:

Public Health, maternal and Child Health, Nutrition, Research designs, environmental health

I confirm that I have read this submission and believe that I have an appropriate level of expertise to confirm that it is of an acceptable scientific standard.

# Response

**Published:** 2024-10-10

**Authors:** Saqib Ali

**Affiliations:** BDS Department, Imam Abdulrahman Bin Faisal University, Dammam, Eastern Province, Saudi Arabia

Dear Reviewer 

 Many thanks for the constructive feedback. We have addressed all the comments and did the modifications accordingly. Please find below the responses to all your comments.

**Reviewer comment: **The presented study briefly encapsulates the essence of your study on the assessment of mental health and psychological distress within the general population of Peshawar, Pakistan, during the COVID-19 pandemic. Here are some suggestions and comments for potential refinement and further consideration:

 1. Introduction: It's concise and effectively sets the stage for the study's relevance. You might want to add a brief note on why Peshawar specifically was chosen, if there's a unique context that makes this study particularly important for the region.

**Author response: **Thank you for your positive feedback on the introduction. We appreciate your suggestion to provide more context on why Peshawar was specifically chosen for this study.

 Peshawar was selected due to its status as a major healthcare hub in Pakistan, hosting several tertiary care hospitals that serve a large and diverse population. The city’s healthcare institutions train a substantial number of dental professionals, including interns and postgraduates, who experience unique challenges and stressors related to their demanding schedules and high patient load. Additionally, factors such as cultural attitudes, lifestyle, and socioeconomic conditions in Peshawar may contribute to sleep disorders in ways that differ from other regions, making it a valuable location for this study.

 We will incorporate these points into the introduction to highlight the significance of studying sleep disorders among dental professionals specifically in Peshawar, providing a stronger rationale for the study’s relevance to the region. Thank you for this helpful suggestion.

**Reviewer comment: **2. Expand Background Context: Provide more background on the mental health impacts of pandemics historically or from recent events prior to COVID-19. This could help frame the significance of your study within a broader context.

**Author response:** Thank you for the suggestion to expand the background context by exploring the mental health impacts of pandemics from a historical perspective. This is a valuable idea, as it can help frame the significance of our study within a broader context.

 Historically, pandemics have had profound impacts on mental health, as observed in past events such as the **Spanish Flu of 1918**, which led to widespread anxiety, grief, and psychological distress due to high mortality rates and societal disruption. Similarly, during the **SARS outbreak in 2003**, studies reported increased levels of depression, anxiety, and post-traumatic stress symptoms among healthcare workers and the general population. Research from the **Ebola outbreak** in West Africa in 2014 also highlighted severe psychological consequences, including stigma, isolation, and an increase in mental health disorders among survivors and healthcare professionals.

 These historical events demonstrate that pandemics often lead to substantial mental health challenges, including heightened stress, anxiety, and depression, due to factors such as fear of infection, prolonged isolation, and socioeconomic strain. By framing the COVID-19 pandemic within this broader historical context, we can underscore the importance of addressing mental health impacts as an integral part of pandemic response and recovery efforts.

 We will incorporate this background into the study to emphasize the relevance of examining mental health impacts during pandemics and provide a richer context for our research. Thank you for this insightful recommendation.

**Reviewer comment: **3. Method: This section is clear about the study's design and tools used for data collection. It could be enhanced by briefly mentioning the rationale behind using the Kessler Psychological Distress Scale specifically, which could help non-specialist readers understand its relevance and validity in this context.

**Author response: **Thank you for your feedback on the methods section. We appreciate your suggestion to include a brief rationale for using the **Kessler Psychological Distress Scale (K10)** in this study.

 The K10 scale was selected due to its strong validity and reliability in measuring psychological distress across diverse populations and its sensitivity to detecting symptoms of anxiety and depression. It consists of 10 questions that assess the frequency of emotional states associated with psychological distress, making it an efficient tool for identifying individuals at risk for mental health disorders. The scale is widely used in public health research due to its ease of administration and its ability to provide a quick, standardized measure of distress levels, which is particularly useful in large-scale studies like ours.

 Including the K10 allows us to accurately quantify levels of psychological distress among participants, enabling comparisons across different groups. This choice is especially relevant for our study, as it provides a validated measure to assess mental health impacts in the context of the pandemic, making the findings more robust and generalizable.

 We will incorporate this explanation into the methods section to clarify the rationale behind choosing the Kessler Psychological Distress Scale, especially for readers who may not be familiar with the tool. Thank you for the valuable suggestion.

**Reviewer comment: **4. The conclusion rightly emphasizes the implications for policy. It could be strengthened by explicitly recommending specific policy measures or interventions that could be implemented based on the study findings.

**Author response: **Thank you for your feedback on the conclusion. We appreciate your suggestion to strengthen it by providing specific policy recommendations based on the study’s findings.

 To make the implications for policy more actionable, we propose to include recommendations for targeted interventions. For example, based on our findings, we can recommend implementing mental health support programs specifically tailored for healthcare professionals, such as stress management workshops, counseling services, and peer support networks within hospitals. Additionally, policies that promote regular screening for psychological distress using tools like the Kessler Psychological Distress Scale could be introduced as part of routine health check-ups for healthcare workers, especially during periods of heightened stress, such as pandemics.

 Further, workplace policies that encourage reasonable work hours, regular breaks, and sufficient leave days can help mitigate stress and improve overall well-being among healthcare staff. These measures, supported by healthcare institutions and policymakers, could help address and prevent psychological distress more effectively, promoting a healthier, more resilient workforce.

 We will incorporate these specific policy recommendations into the conclusion to provide a more concrete foundation for translating our findings into practical interventions. Thank you for your helpful suggestion to enhance this section.

**Reviewer comment: **5. Address Bias and Generalizability in your limitations. More thoroughly discuss how the method of survey distribution (online) might limit the generalizability of the findings. For instance, does this method potentially exclude older populations or those without internet access?

**Author response:** Thank you for highlighting the importance of addressing potential biases and limitations related to survey distribution. We agree that discussing how the online survey method may impact the generalizability of our findings is essential.

 In the limitations section, we will expand on the potential biases introduced by using an online survey format. Specifically, we recognize that relying on online distribution may exclude certain demographics, such as older individuals who may not be as comfortable with digital technology or those without consistent internet access. This limitation could lead to an underrepresentation of these groups, potentially skewing our findings towards younger, more tech-savvy participants. Additionally, the online format may introduce selection bias, as those experiencing higher levels of psychological distress may be more inclined to participate in a survey on this topic.

 To improve generalizability in future studies, we recommend complementing online surveys with alternative distribution methods, such as paper surveys or in-person interviews, especially in settings where internet access is limited. By acknowledging these limitations, we aim to provide a balanced interpretation of our findings and offer suggestions for future research to mitigate these biases.

 Thank you for your valuable suggestion to enhance this aspect of our discussion on study limitations.

# Reviewer report: Reviewer response for version 1

**DOI:** 10.5256/f1000research.159438.r272714

**Published:** 2024-05-31

**Authors:** Niaz Ali

**Affiliations:** 1 College of Medicine, Shaqra University, Shaqra, Riyadh Province, Saudi Arabia

The study is done extensively and is of interest but it needs major revisions so it can be of greater benefit for the community. Please find my detailed comments below. All the very best.

 1. Please check the manuscript for grammatical errors and typos. Some sentences are too long to follow.

 2. Consider refining the use of passive voice where possible, to active voice, which can make the abstract more engaging and easier to read.

 3. Ensure consistency in statistical reporting, for example, always use the same number format for p-values (e.g., p < 0.001 or p = 0.001).

 4. If space allows in the body of the article, it would be valuable to include more detailed analysis or discussion about the impact of COVID-19 on different age groups or between genders. This can provide deeper insights into which sub-groups are most vulnerable.

 5. In the recommendations, besides general calls for policy action, suggest specific mental health resources or programs that have been effective in similar contexts.

 6. Ensure all references are up to date and correctly formatted, and check if the latest statistics or studies might provide more recent insights into the COVID-19 impact on mental health globally and locally.

 7. The abstract provides a solid overview of your research and its importance to public health discourse in Pakistan. By tightening the focus a bit more on specific actionable insights and perhaps providing a bit more context about the tools and demographic influences, it can serve as a robust entry point for readers into the study's full findings.

Is the work clearly and accurately presented and does it cite the current literature?

Yes

If applicable, is the statistical analysis and its interpretation appropriate?

Yes

Are all the source data underlying the results available to ensure full reproducibility?

Yes

Is the study design appropriate and is the work technically sound?

Yes

Are the conclusions drawn adequately supported by the results?

Yes

Are sufficient details of methods and analysis provided to allow replication by others?

Yes

Reviewer Expertise:

Social Science, Pharmacology, Medical Education, Dental Education, Psychological,

I confirm that I have read this submission and believe that I have an appropriate level of expertise to confirm that it is of an acceptable scientific standard, however I have significant reservations, as outlined above.

# Response

**Published:** 2024-10-10

**Authors:** Saqib Ali

**Affiliations:** BDS Department, Imam Abdulrahman Bin Faisal University, Dammam, Eastern Province, Saudi Arabia

Dear Reviewer

 Many thanks for the constructive feedback. We have addressed all the comments suggested and improved the manuscript per your suggestions which are as below:

 The study is done extensively and is of interest but it needs major revisions so it can be of greater benefit for the community. Please find my detailed comments below. All the very best.

 1. Please check the manuscript for grammatical errors and typos. Some sentences are too long to follow.

**Response:** Thank you for your feedback regarding grammatical errors and sentence structure. We will thoroughly review the manuscript for any typos and grammatical mistakes to ensure clarity and accuracy. Additionally, we will revise overly long sentences to improve readability and ensure that the flow of ideas is smooth and easy to follow.

 In particular, we will focus on breaking down complex or lengthy sentences into shorter, more concise ones where possible. This will help make the content more accessible to readers and improve the overall presentation of the manuscript.

 2. Consider refining the use of passive voice where possible, to active voice, which can make the abstract more engaging and easier to read.

**Response: **We will review the abstract carefully and rephrase sentences in active voice wherever appropriate. For example, rather than saying, "Data was collected from participants," we can change it to, "We collected data from participants." This adjustment will help create a more direct and dynamic tone, making the content easier to follow and more compelling.

 3. Ensure consistency in statistical reporting, for example, always use the same number format for p-values (e.g., p < 0.001 or p = 0.001).

**Response:** Thank you for pointing out the need for consistency in statistical reporting. We will review the manuscript to ensure that all statistical values, particularly p-values, follow a uniform format throughout (e.g., consistently using "p < 0.001" or "p = 0.001"). This will improve the clarity and professionalism of the presentation.

 4. If space allows in the body of the article, it would be valuable to include more detailed analysis or discussion about the impact of COVID-19 on different age groups or between genders. This can provide deeper insights into which sub-groups are most vulnerable.

**Response: **Thank you for your suggestion to expand the analysis on the impact of COVID-19 across different age groups and genders. We agree that a more detailed examination of how various demographic sub-groups are affected could provide valuable insights into which populations may be more vulnerable to psychological distress.

 If space permits in the body of the article, we will include an analysis that breaks down the mental health impacts of COVID-19 by age and gender. This could involve examining whether certain age groups, such as older adults or young adults, experience higher levels of anxiety or depression. Additionally, we can explore any gender-based differences in psychological responses, which may help identify specific needs for targeted intervention strategies.

 5. In the recommendations, besides general calls for policy action, suggest specific mental health resources or programs that have been effective in similar contexts.

**Response: **Thank you for your suggestion to include specific mental health resources and programs in our recommendations. We agree that mentioning effective programs from similar contexts can provide actionable insights for policymakers and healthcare providers. We will update it.

 6. Ensure all references are up to date and correctly formatted, and check if the latest statistics or studies might provide more recent insights into the COVID-19 impact on mental health globally and locally.

**Response: **Thank you for your feedback on ensuring reference accuracy and currency. We will review all references to confirm they are up-to-date and correctly formatted. Additionally, we will search for the latest studies and statistics on COVID-19's global and local mental health impact to provide the most current insights in our discussion.

 7. The abstract provides a solid overview of your research and its importance to public health discourse in Pakistan. By tightening the focus a bit more on specific actionable insights and perhaps providing a bit more context about the tools and demographic influences, it can serve as a robust entry point for readers into the study's full findings.

**Response: T**hank you for your feedback. We will tighten the abstract’s focus on specific actionable insights and add brief context about the tools used and demographic influences. These updates will create a clearer and more engaging entry point for readers into our study's findings.
